# Allergic Reactions to COVID-19 Vaccination in High-Risk Allergic Patients: The Experience of Trieste University Hospital (North-Eastern Italy)

**DOI:** 10.3390/vaccines10101616

**Published:** 2022-09-27

**Authors:** Francesca Larese Filon, Ilaria Lazzarato, Emilia Patriarca, Thomas Iavernig, Alberto Peratoner, Giuseppe Perri, Giuliano Ponis, Giulio Rocco, Luca Cegolon

**Affiliations:** 1 Unit of Occupational Medicine, Department of Medical Surgical & Health Sciences, University of Trieste, 34129 Trieste, Italy; 2 Unit of Occupational Medicine, University Health Agency Giuliano-Isontina (ASUGI), 34129 Trieste, Italy; 3 Accident & Emergency, University Health Agency Giuliano-Isontina (ASUGI), 34129 Trieste, Italy; 4 Directorate Office, Cattinara Hospital, University Health Agency Giuliano-Isontina (ASUGI), 34149 Trieste, Italy; 5 Hospital Pharmacy, Giuliano Area, University Health Agency Giuliano-Isontina (ASUGI), 34128 Trieste, Italy; 6 Public Health Department, University Health Agency Giuliano-Isontina (ASUGI), 34128 Trieste, Italy

**Keywords:** COVID-19, vaccination, allergy, anaphylaxis, reactions, polyethylene glycol, polysorbate, drug allergy

## Abstract

**Background**. Allergic patients may develop reactions following COVID-19 vaccination more frequently than non-allergic individuals. The aim of our study was to assess the risk of reactions in high-risk allergic patients vaccinated for COVID-19 at the University Health Agency Giuliano-Isontina (ASUGI) of Trieste (northeastern Italy). **Methods**. Patients were considered at high risk for allergic reactions in case of: prior anaphylactic reaction to any drug/vaccine; multiple drug allergy; intolerance to polyethylene glycol (PEG) or polysorbate 80 (PS80) containing drugs; and mast cell disorders. High-risk allergic patients were immunized in hospital by a dedicated allergy team supported by resuscitation staff. Patients were interviewed over the phone one month after vaccination to complete a structured questionnaire investigating signs and symptoms developed after immunization. **Results**. From March 2021 to February 2022, 269 patients with a history of severe allergic reactions were assessed, of whom 208 (77.3%) eventually received COVID-19 vaccination, 50 (18.6%) refused to be immunized, 10 (3.7%) were deferred for medical reasons and one was declared exempted due to testing positive for PS80. Mild reactions (urticaria, angioedema, rhinitis, erythema) to COVID-19 vaccines were reported by 30.3% of patients, 8.7% within 4 h and 21.6% > 4 h after immunization. No anaphylactic events were observed. Although they were 80 times (3.8%) more prevalent than in COVID-19 vaccinees from the general population (0.047%), vaccine allergic reactions in high-risk patients were mainly mild and late, more likely affecting women (OR = 3.05; 95% CI 1.22–7.65). **Conclusions**. High-risk allergic patients with urticaria and angioedema may experience mild flare-ups of mast cell activation-like symptoms following COVID-19 vaccination, supporting antihistamine premedication before vaccination and to be continued for one week afterwards.

## 1. Introduction

In Italy, the vaccination campaign against COVID-19 started on 27 December 2020. COVID-19 vaccination was made mandatory for all health care workers (HCWs) from April 2021 and for individuals older than 50 from January 2022 onward. Unvaccinated HCWs were suspended from work or re-assigned to work tasks not entailing patient contact. 

At the University Health Agency Giuliano-Isontina (ASUGI), the vaccination campaign against COVID-19 started with Comirnaty (Pfitzer-BioNTech, BNT162b2) and Vaxzevria (Astra Zeneca), and from May 2021 Spikevax (Moderna) was introduced. The risk of allergic reactions to COVID-19 vaccines was immediately a matter of concern due to the exclusion of allergic patients in phase-three clinical studies on Comirnaty [1] and patients with a history of allergic reaction to any component of COVID-19 vaccine or an allergy to other vaccines in Spikevax trials [2,3]. 

The risk of anaphylaxis after vaccination with Comirnaty was 11.1 per million injections in an initial report from the USA [4] and 4.7 cases per million doses in a greater population of vaccinees [5], an estimate 5–10 times higher than that reported for other non-COVID-19 vaccines [6]. 

Polyethylene glycol (PEG) and tromethamine for mRNA vaccines and polysorbate 80 (PS80) for Vaxzevria were immediately indicted as possible culprit allergens for allergic reactions [7] and specific recommendations were suggested to screen patients with a history of allergic reactions before immunizing them with COVID-19 vaccines [8,9,10].

In view of the above, the aim of this study was to assess allergic reactions following COVID-19 vaccination among high-risk allergic patients in ASUGI Trieste.

## 2. Materials and Methods

The present study was conducted according to the guidelines of the Declaration of Helsinki and approved by the ethical committee of the Friuli–Venezia Giulia region (CEUR- 2020-Os-072) on 16 April 2020. Patient consent was waived since, according to Italian privacy law (Legislative Decree 101/2018, D.Lgs 101/2018), patients’ data routinely collected by the Italian National Health Service (NHS) can be used for scientific purposes within the scope of approved studies/protocols, provided sensitive information is anonymized.

At the Unit of Occupational Medicine of Trieste Teaching Hospital, an allergy task force (ATF) was set up to assess the risk of allergic reactions to COVID-19 vaccines in high-risk allergic patients and their eligibility for vaccination within the catchment area of the health district of Trieste. General practitioners and health care staff at COVID-19 vaccination centers referred high-risk individuals with history of severe allergic reactions for immunization under special medical supervision. 

In accordance with the open literature [7,9] and the guidelines of the Italian Association of Allergologists and Immunologists [11], patients with sensitivity to aeroallergens, latex, or contrast media, as well as patients with single-drug or venom allergy, were immunized under standard conditions and monitored for 60 min after vaccination. Patients meeting the following criteria underwent medical assessment by the ATF:

- History of allergic or anaphylactic reaction to multiple oral or injectable drugs or vaccines;
- History of idiopathic anaphylaxis;
- History of mast cell disorders;
- History of chronic urticaria;
- History of uncontrolled asthma.

Multiple drug allergy was defined as a history of hypersensitivity to more than 1 drug group. An allergy workout was set up to evaluate drug intake in patients and their tolerance to excipients such as PEG and PS80. 

Patients were considered at high risk of allergic reactions in case of:

- Prior anaphylactic reaction to any drug or vaccine;
- Multiple drug allergy without tolerance to PEG- or PS80-containing drugs;
- Mast cell disorders;
- Patients with uncontrolled asthma who had to be treated for their condition to achieve satisfactory control of their symptoms before being vaccinated for COVID-19.

Patients considered at high risk of allergic reaction were immunized in a hospital setting and monitored for 1–2 h following vaccination by a dedicated allergy team, including resuscitation medical staff. Although patients had to continue their usual anti-allergic therapy post immunization, including antihistamines [7], premedication was not recommended before any COVID-19 vaccine dose [11]. Upon administration of the second and third dose of the COVID-19 vaccine, health care staff asked vaccinees to report any reactions to previous doses. One month after immunization, patients were contacted by the ATF over the phone to complete a structured survey questionnaire investigating the signs and symptoms developed after any COVID-19 vaccine dose. Early adverse reactions were defined as those occurring within minutes to 4 h after vaccine administration; late reactions were defined as those developing >4 h after immunization [7,12]. Local reactions at the site of the injections were not considered in the analysis. Definitions of some immunological conditions and technical terms used across this manuscript can be viewed in Supplementary Table S1 [13,14,15,16,17].

## 3. Statistical Analysis

The data of patients developing reactions were compared with those not developing reactions. 

Continuous data were reported as median (interquartile range, IQR) and mean ± standard deviation and compared using the Mann–Whitney test. Categorical data were compared by chi-square statistics.

Prevalence of allergic reactions in the study population was compared to that in the general population of the same area (Provinces of Trieste and Gorizia) obtained from regional surveillance system for reactions to COVID-19 vaccines.

A multivariable logistic regression was used to investigate risk factors for allergic reactions post COVID-19 vaccination, selecting terms significant at univariable analysis. 

No variable had missing values. A *p*-value < 0.05 was set as a threshold for statistical significance.

Statistical analysis was performed using STATA 17 (STATA Corp, University City, TX, USA).

## 4. Results

**Figure 1:**
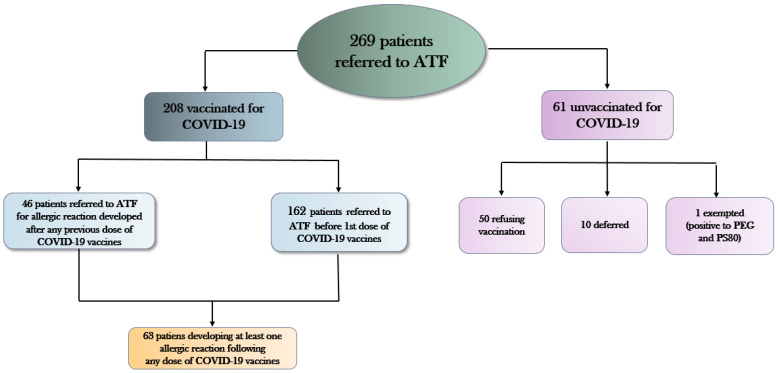
Patients with history of allergic reactions referred to the allergy task force (ATF) of Trieste from March 2021 to February 2022. PEG = polyethylene glycol; PS80 = polysorbate 80.

Two hundred and sixty-nine patients with history of allergic reactions were assessed by the ATF of Trieste from March 2021 to February 2022. As can be seen from Figure 1, among the latter high-risk allergic group:

- 208 (77.3%) patients received COVID-19 vaccination;
- 50 (18.6%) patients refused to be immunized
- 10 (3.7%) patients were deferred for medical reasons (uncontrolled asthma, ongoing urticarial reactions, other acute allergic diseases);
- 1 patient tested positive for PEG and PS80 and was declared exempt from mandatory vaccination against COVID-19.

As can be seen from Table 1, high-risk allergic patients of ASUGI Trieste were predominantly females (79.6%) and had a median age of 55 years (IQR: 45–64 years). Moreover, 53.5% (=144/269) of patients had a history of drug allergy and 18.2% (=49/269) reported drug anaphylaxis. Food allergy was reported by 69 (25.6%) patients and 2.6% (=7/269) with food anaphylaxis. Eighteen patients underwent skin tests for PEG, none of whom were positive, whereas three were tested for PS80 with only one turning up positive. 

**Figure 2:**
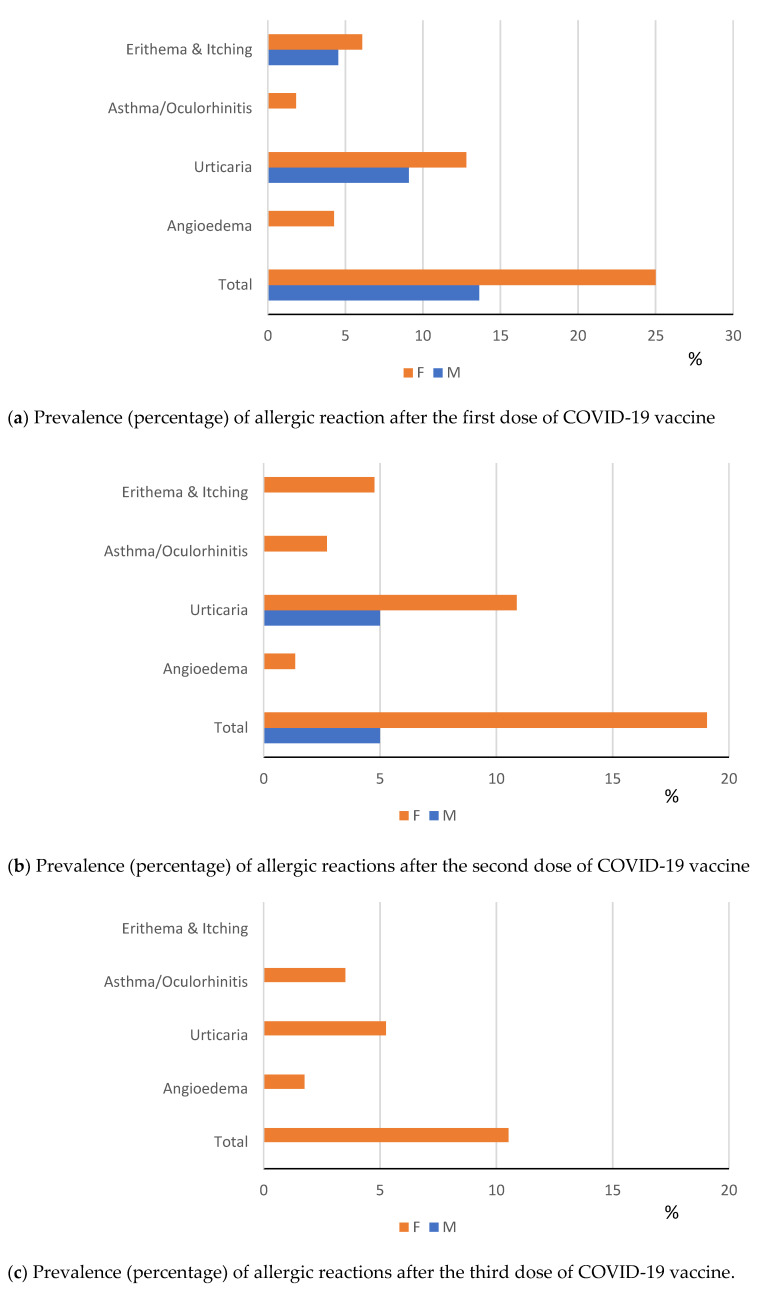
(**a**–**c**) Distribution of allergic reactions by dose of COVID-19 vaccine administered. M = Males; F = Females.

One hundred and twenty patients were vaccinated under special medical supervision. Mild allergic reactions (urticaria, angioedema, rhinitis and erythema) post COVID-19 vaccinations occurred in 30.3% patients, 8.7% early (within 4 h) and 21.6% late (>4 h) after immunization. Figure 2a–c show the distribution of the allergic reactions by dose of COVD-19 vaccine received.

Table 2 displays the results of univariable as well as multivariable logistic regression analysis investigating risk factors for allergic reactions to COVID-19 vaccinations. As can be seen, females were more likely to develop an allergic reaction (OR = 3.05; 95% CI: 1.22; 7.65), whereas patients withhistory of allergic reactions to drugs had a significantly lower risk of reaction (OR = 0.30; 95% CI: 0.15; 0.58).

The details of the adverse reactions in each patient by COVID-19 vaccine dose in each patient can be viewed in Table 3. As can be seen, no allergic reaction within or after 4 h since COVID-19 vaccination involved two districts simultaneously (skin as well as respiratory system). Among the study group:

- 29 patients developed an allergic reaction only to the first vaccine dose (orange coloured), of whom 8 were referred to ATF medical examination before dose 1 and 21 between dose 1 and dose 2.
- 11 patients developed an allergic reaction only after the second vaccine dose (green coloured), of whom 3 were referred to ATF medical examination before dose 1 and 8 between dose 1 and dose 2.
- 3 patients developed an allergic reaction only to the third dose (yellow coloured), all referred to ATF medical examination before dose 1.
- 17 patients reacting to the first vaccine dose reacted also to the second (grey colored), of whom 5 were referred to ATF medical examination before the first dose and 12 between dose 1 and dose 2;
- 2 patients referring to AFT medical examination before the first dose did not develop any allergic reaction to the first dose, but reacted after both dose 2 as well as dose 3 (pink colored);
- 1 patient referring to ATF between first and second dose reacted against both dose 1 as well as dose 2;
- No patient was referred to ATF medical examination after the second dose of COVID-9 vaccine.

Table 4 reports the aggregated adverse and allergic reactions to different COVID-19 vaccines in the general population of the Trieste–Gorizia catchment area, by dose administered. The overall prevalence of allergic reactions was 0.047% (=333/706,573 doses administered in 2021) and only 8 vaccinees were recorded in the Italian national surveillance system for adverse vaccine events. No cases of anaphylaxis occurred and reactions included urticarial, angioedema, itching, asthma and rhinitis events. Thirty-three cases were classified as “severe reactions” requiring treatment and medical support. Considering only official data, the prevalence of allergic reactions was significantly higher (*p* < 0.001) in vaccinated patients with history of severe allergy (3.8% = 8/208) compared to that in COVID-19 vaccinees from the general population (0.047%).

## 5. Discussion

Within the catchment area of ASUGI Trieste, 706,573 doses of COVID-19 vaccines were administered during calendar year 2021 to a total population 373,896 local residents. Vaccine hesitancy was immediately considered from the beginning of the campaign and an ATF was set up by ASUGI Trieste with the aim to evaluate patients with a history of multiple allergies and those with previous reactions to COVID-19 vaccines. In our cohort, 269 subjects were assessed because they were considered at higher risk of allergic reaction, 46 of whom already had an allergic reaction following a previous dose of COVID-19 vaccine. 

Out of 208 patients vaccinated against COVID-19, none developed anaphylaxis or severe allergic reactions, whereas 63 (30.3%) reported an allergic reaction, mainly urticarial—with wide difference by sex (16.5% in females vs. 9.1% in males)—followed by angioedema, erythema/itching and a few cases of asthma/oculo-rhinitis. Vaccine reactions were overall mild and in a third of cases immediate, i.e., developing within four hours of vaccination. However, the prevalence of allergic reactions in immunized patients with history of severe allergy (3.8%) was 80 times that of COVID-19 vaccinees from the general population (0.047%) of the same catchment area.

The higher proportion of allergic reactions to COVID-19 vaccines in females has already been reported ^18^ and is also consistent with the prevalence of allergic disease and particularly drug allergy in females [18,19,20,21,22,23]. Among the study group, food allergy was reported by 28.2% patients, drug allergy by 53.5% and 18.2% had a history of anaphylactic reaction to medications. Lower figures on food allergy (15.9%) and multiple drug allergy (32.9%) were found in a prospective cohort study conducted at Sheba Medical Centre (Tel Aviv, Israel) from 27 December 2020 to 22 February 2021 on 8102 patients with allergy history [20]. By contrast, the prevalence of food allergy in the general population is estimated to be <5% [14,21,24,25] and severe or multiple drug allergies is <1% [26].

In a study using US data from the Vaccine Adverse Event Reporting System (VAERS), out of 14,611 patients developing reactions, those with a history of allergy were more likely to develop immediate reactions after COVID-19 vaccination, especially vaccinees with history of anaphylaxis (OR 7.16; 95% CI 3.50–14.65) [13]. Likewise, in another analysis on VAERS data from 55 USA states between 14 December 2020 and 5 February 2021, out of 36,819,212 doses of COVID-19 vaccines administered as of 5 February 2021, the incidence of anaphylaxis among 12,630 COVID-19 vaccine recipients was 4.2 per million doses, with a risk of 1.85% (95% CI: 1.44%; 2.25%) among those with previous allergies and 7.91% (95% CI: 3.33%; 12.49%) among those with history of anaphylaxis [27]. In a further online survey on 1808 Polish respondents, 1707 of whom received two doses of Comirnaty, whilst reactions were far more likely after the second dose and among high-risk allergic patients, the majority of these reactions were mild and did not preclude the completion of the vaccination cycle [28].

In the present study, in case of a previous reaction to multiple drugs containing PEG or PS80 and intolerance to any drugs containing the latter two preservatives, skin tests were performed (N = 18 for PEG and N = 3 for PS80 and macrogol), finding only one positive reaction to PS80 in a patient with history of generalized reaction to PEG (5.6%). The utility of skin tests against the latter two preservatives is debatable although recommended by the Italian and International Societies for Allergic Diseases [7,11,29,30]. However, in a US study on 80 consecutive patients reporting allergic reactions to m-RNA COVID-19 vaccines, skin testing did not influence the tolerance against the second dose, which was well-received by the majority of vaccinees (N = 70) [31]. However, it is worth mentioning that COVID-19 vaccines contain several further excipients rarely inducing allergy, such as aluminum hydroxide, disodium EDTA, Matrix M™, Matrix-A and Matrix-C particles [32]. The latter excipients are not only included in vaccine preparations but can also be present in various everyday items, which could potentially sensitize users [33].

In our study, high-risk allergic patients were monitored for an extended time (at least 1 h) following vaccination and no cases of anaphylaxis were observed. 

Allergic reactions to COVID-19 vaccines can be early, delayed or late [34,35]. Rapid reactions are mediated by IgE antibodies against a vaccine component, or via another mechanism activating mast cells [20] as a complement activation-related pseudoallergy (CARPA), already described for liposomes [29]. Other reactions include late onset of urticaria, eczema, erythema and itching [36,37]. Late-onset or delayed reactions following COVID-19 vaccination may include urticaria, eczema, erythema or itching hours to days after immunization, or late-onset local injection site reactions or swelling at the site of dermal fillers, developing several days after immunization [35,36,37]. 

In our study, 8.7% of patients reporting allergic reactions (mainly urticaria and angioedema) within 4 h after immunization were treated with antihistamine and cortisone, whereas 21.6% developed reactions (mainly urticarial) >4 h after vaccination. Likewise, in a Belgian survey by telephone interviews, 25 (11.3%) immediate reactions were observed among 221 doses of m-RNA or adeno-viral vector vaccines administered to 196 patients at risk of immediate hypersensitivity reactions at the university hospital of Leuven (Belgium) [23]. By contrast, in the above Israeli study from Sheba Medical Centre in Tel Aviv, only 9 (2%) out of 429 highly allergic patients developed immediate reactions, three (0.7%) of which were anaphylactic [20].

In the present study, only 8 (3.8%) patients had a relevant reaction according to the Italian Database for Adverse Event Following Immunization, whereas for other patients adverse reactions were mild and therefore were neglected. 

The prevalence of skin reactions was 21.6% in the present study, a rather high figure not far from that (15%) reported for skin eruption and itching in the above Israeli study from Sheba Medical Centre [20]. Ieven et al. [23] in 2022 also reported that 13.2% of late reactions had mast cell activation-like symptoms. However, the latter reactions were mild and self-limited in the present study. 

### Strengths and Limitations 

A strength of our study is the thorough and in-depth clinical assessment and follow up of each patient, thereby maximizing a reliable detection of allergic reactions to COVID-19 vaccination. Investigating vaccinees over the phone allowed us to identify symptoms in a detailed fashion, finding a very low incidence of severe reactions and a rather high incidence of mild and late allergic symptoms following immunization. Some published studies on the reactions to COVID-19 vaccines rely on health registries collecting adverse effects rather than actually following up vaccinees individually [2,20,38].

However, the present study also has some limitations. Patients reported allergic reactions after vaccination during a telephone interview, an approach potentially affected by recall bias and an overestimation of the reactions, since allergic people also tend to report very mild side effects after vaccination (nocebo effect). However, it is also well known that the Database for Adverse Event Following Immunization underestimates reactions because only significant or severe reactions tend to be reported.

Only in case of suspicion of allergy to PS80 or PEG skin tests were performed in the present study, hence a limited number of patients were tested, with only one subject resulting positive for PS80. As already mentioned, the utility of these skin tests and the relevance of sensitization to these two excipients is still under debate [31]. 

Furthermore, although skin tests against vaccines themselves were not performed, the assessment of allergy to COVID-19 vaccines by intradermal testing with the entire vaccine should be systematically considered, offering tailored immunization protocols (e.g., alternative vaccine, premedication, desensitization, etc.) to patients at high risk of allergic reactions [39]. 

## 6. Conclusions

The prevalence of mild yet late allergic reactions in immunized patients with a history of severe allergy was 80 times that in COVID-19 vaccinees’ from the general population. The use of antihistamine as premedication, to be continued for one week following immunization, may be considered to prevent these adverse reactions in selected patients appropriately screened.

## Figures and Tables

**Table 1:** Distribution of the study population. Number (N); column percentage (Col %); mean (M) ± standard deviation (SD); median and interquartile range (IQR). PS80 = Polysorbate 80.

FACTORS				Males N (Col %)	Females N (Col %)	Total N (Col %)	*p*-Value *
**Total patients**				55 (20.4)	214 (79.6)	269 (100)	
**Age (years)**		**Mean ± SD**		53.7 ± 17.7	54.1 ± 14.4	54.0 ± 15.1	0.439
		**Median (IQR)**		53 (41–66)	55 (45–64)	55 (45–64)	0.893
**Atopic dermatitis**		**No**		53 (86.4)	213 (93.9)	40 (94.4)	0.482
		**Yes**		2 (3.6)	13 (6.1)	15 (5.6)	
**Allergy to inhalants**		**No**		37 (66.3)	128 (59.8)	165 (61.33)	0.072
		**yes**	**Others**	17 (30.9)	86 (40.2)	103 (38.3)	
			**Anaphylaxis**	1 (1.8)	0 (0)	1 (0.37)	
**Food allergy**		**No**		40 (72.7)	153 (71.5)	193 (71.8)	0.159
		**Yes**	**Others**	13 (23.6)	56 (26.2)	69 (25.6)	
			**Anaphylaxis**	2 (3.6)	5 (2.3)	7 (2.6)	
**Drug allergy**		**No**		21 (38.2)	55 (25.7)	76 (28.3)	0.159
		**Yes**	**Others**	24 (43.6)	120 (56.1)	144 (53.5)	
			**Anaphylaxis**	10 (18.2)	39 (18.2)	49 (18.2)	
**Hymenoptera allergy**		**No**		53 (96.4)	200 (93.5)	253 (94.0)	0.001
		**Yes**	**Others**	1 (1.8)	11 (5.1)	12 (4.5)	
			**Anaphylaxis**	1 (1.8)	3 (1.4)	4 (1.5)	
**Latex allergy**		**No**		54 (98.2)	178 (83.2)	232 (86.2)	0.001
		**Yes**	**Others**	0 (0)	36 (16.8)	36 (13.4)	
			**Anaphylaxis**	1 (1.8)	0 (0)	1 (0.4)	
**Contrast medium** **allergy**		**No**		50 (90.9)	199 (94.0)	249 (92.5)	0.459
		**Yes**	**Others**	3 (5.4)	5 (2.3)	8 (3.0)	
			**Anaphylaxis**	2 (3.6)	10 (4.7)	12 (4.5)	
**Asthma**		**No**		48 (85.4)	166 (77.6)	?	0.046
		**Yes**	**Controlled**	8 (14.6)	44 (20.6)	52 (19.3)	
			**Uncontrolled**	0 (0)	4 (1.9)	4 (1.5)	
**Chronic urticaria**		**No**		53 (96.4)	96 (91.6)	249 (92.6)	0.229
		**Yes**		2 (3.6)	18 (8.4)	20 (7.4)	
**Angioedema**		**No**		50 (89.9)	201 (93.9)	251 (93.3)	0.425
		**Yes**		5 (9.1)	13 (6.1)	18 (6.7)	
**Idiopathic anaphylaxis**		**No**		53 (89.9)	201 (98.6)	264 (98.1)	0.274
		**Yes**		2 (3.6)	3 (1.4)	5 (1.9)	
**Suspect Macrogol** **allergy**		**No**		51 (92.7)	206 (86.3)	257 (95.5)	0.257
		**Yes**		4 (7.3)	8 (3.7)	12 (4.5)	
**Suspect PS80 allergy**		**No**		54 (98.2)	211 (98.6)	265 (98.5)	0.820
		**Yes**		1 (1.8)	3 (1.4)	4 (1.5)	
**Allergy test**		**No**		50 (89.9)	198 (92.5)	248 (92.2)	0.195
		**Yes**	**Macrogol**	4 (7.3)	14 (6.5)	18 (6.7)	
			**Macrogol + PS80**	1 (1.8)	2 (0.9)	3 (1.1)	
**Positive reactions to Macrogol**				0	0	0	NA
**Positive reactions to PS80**		**No**		0	2 (0.9)	2 (0.75)	NA
		**Yes**		1 (1.8)	0 (0)	1 (0.4)	
**COVID-19 vaccination under** **medical supervision**		**No**		30 (54.6)	119 (54.6)	149 (55.4)	0.805
		**Yes**		25 (45.4)	95 (44.4)	120 (44.6)	
**COVID-19** **vaccination status**		**Vaccinated (1+ doses)**		44 (80.0)	164 (76.6)	208 (77.3)	0.030
		**Refusing vaccination**		5 (9.1)	44 (20.6)	49 (18.2)	
		**Vaccination deferred before dose 1**		4 (7.3)	6 (2.8)	10 (3.7)	
		**Exempted (allergy to PEG and PS80)**		1 (1.8)	0 (0)	1 (0.4)	
**Allergy consultancy** **before COVID-19** **vaccination**		**No**		4 (3.3)	43 (19.3)	47 (17.9)	0.033
		**Yes**		51 (92.7)	171 (80.7)	222 (83.1)	
**Reactions to** **COVID-19** **vaccine**	**No**			32 (72.7)	92 (56.1)	124 (59.6)	0.046
	**Yes**	**Any type of reaction**		12 (27.3)	72 (43.9)	84 (40.4)	
		**At least one** **allergic** reaction **	**Total**	7 (15.9)	56 (34.2)	63 (30.3)	0.019
			**<4 h since vaccination**	0	18 (11.0)	18 (8.7)	0.080
			**>4 h since vaccination**	7 (15.9)	38 (23.1)	45 (21.6)	

* chi-square test (for categorical terms) or Wilkoxon test (for median differences) or ANOVA (for mean differences). ** Urticaria or angioedema or rhinitis or erythema (p compared vs no reactions)

**Table 2:** Univariable and multivariable logistic regression analysis on risk factors for allergic reactions following COVID-19 vaccinations. Odds ratios unadjusted (ORs) and adjusted (aORs) with 95% confidence intervals (95% CI).

FACTORS			UNIVARIABLE		MULTIVARIABLE	
			OR (95% CI)	*p*-Value	aOR (95% CI)	*p*-Value
**Age (years, linear term)**			0.99 (0.97; 1.01)	0.286		
**Sex**	**Males**		reference		reference	
	**Females**		2.74 (1.14; 6.54)	0.023	**3.05 (1.22; 7.65)**	**0.017**
**Atopic dermatitis**	**No**		reference			
	**Yes**		1.34 (0.38; 4.73)	0.653		
**Inhalant allergy**	**No**		reference			
	**Yes**		1.31 (0.72; 2.39)	0.375		
**Food allergy**	**No**		reference			
	**Yes**		0.91 (0.47;1.76)	0.771		
**Drug allergy**	**No**		reference		reference	
	**Yes**		**0.33 (0.18; 0.62)**	**0.001**	**0.30 (0.15; 0.58)**	**<0.001**
**Venom allergy**	**No**		reference			
	**Yes**		1.34 (0.38; 4.74)	0.653		
**Latex allergy**	**No**		**reference**		reference	
	**Yes**		**2.25 (1.00; 5.07)**	**0.050**	1.81 (0.77; 4.24)	0.172
**Contrast medium** **allergy**	**No**		reference			
	**Yes**		0.75 (0.23; 2.42)	0.633		
**Asthma**	**No**		reference			
	**Yes**	**Controlled**	1.15 (0.10; 12.9)	0.910		
		**Uncontrolled**	0.98 (0.46; 2.01)	0.910		
**Chronic urticaria**	**No**		reference			
	**Yes**		0.19 (0.02; 1.56)	0.123		
**Angioedema**	**No**		reference			
	**Yes**		0.76 (0.20; 2.89)	0.682		
**Number of allergic reactions (linear term)**			0.91 (0.68; 1.22)	0.534		
**Vaccine type**	**Comirnaty**		*reference*			
	**Spikevax**		0.96 (0.39; 2.31)	0.957		
	**Vaxzevria**		1.83 (0.29; 11.4)	0.855		

**Table 3:** Allergic reactions to COVID-19 vaccination, reported by high-risk allergic patients of ASUGI Trieste. M = males; F = females.

Patient Number	Sex	Age(years)	History of anaphylaxis	Skin Test	I dose			II dose			III dose			Vaccine type by dose administered		
					Allergic symptoms		Therapy	Allergic symptoms		Therapy	Allergic symptoms		Therapy			
					<4 h	>4 h		<4 h	>4 h		<4 h	>4 h		I Dose	II Dose	III Dose
1	F	53				Urticaria			Urticaria					Comirnaty	Spikevax	
2	F	34				Itching, erythema			Erythema					Comirnaty	Comirnaty	
3	F	52			Urticaria			Urticaria		Cortisone and Antihistamine				Comirnaty	Comirnaty	
4	F	50			Angioedema				Urticaria	Antihistamine				Spikevax	Comirnaty	
5	F	72				Itching	Cortisone and Antihistamine		Itching	Cortisone and Antihistamine				Comirnaty	Comirnaty	
6	F	62				Urticaria	Antihistamine		Urticaria	Antihistamine				Comirnaty	Comirnaty	
7	F	56				Erythema	Antihistamine		Erythema					Spikevax	Spikevax	Spikevax
8	F	65										Oculorinitis		Spikevax	Spikevax	Spikevax
9	F	64							Urticaria	Cortisone and Antihistamine				Comirnaty	Comirnaty	Comirnaty
10	F	49										Angioedema		Comirnaty	Comirnaty	Comirnaty
11	M	70				Urticaria			Urticaria					Comirnaty	Comirnaty	Comirnaty
12	F	63							Urticaria	Cortisone and Antihistamine		Urticaria	Cortisone and antihistamine	Spikevax	Spikevax	Spikevax
13	F	78				Urticaria	Antihistamine							Vaxzevria	Vaxzevria	Spikevax
14	F	45							Urticaria	Antihistamine	Urticaria		Antihistamine	Spikevax	Spikevax	Spikevax
15	F	28	Yes (drugs)		Erythema		Cortisone ed Antihistamine							Comirnaty		
16	F	30			Itching		Cortisone							Comirnaty	Comirnaty	Spikevax
17	F	56			Angioedema		Cortisone							Comirnaty		
18	F	50				Urticaria	Antihistamine							Comirnaty	Comirnaty	Spikevax
19	F	25										Oculorinitis		Comirnaty	Comirnaty	Comirnaty
20	F	70			Angioedema		Cortisone ed Antihistamine							Vaxzevria	Comirnaty	Comirnaty
21	F	74				Erythema								Vaxzevria	Comirnaty	Spikevax
22	F	68				Urticaria	Cortisone ed Antihistamine					Urticaria	Cortisone	Comirnaty	Comirnaty	Comirnaty
23	M	62				Urticaria								Vaxzevria	Comirnaty	
24	F	42			Angioedema		Cortisone and Antihistamine							Spikevax		
25	F	51	Yes (drugs)		Urticaria		Antihistamine							Comirnaty		
26	F	45							Urticaria					Comirnaty	Comirnaty	
27	F	57				Urticaria	Antihistamine							Comirnaty	Comirnaty	
28	F	44			Urticaria		Cortisone	Urticaria		Cortisone				Vaxzevria	Comirnaty	
29	F	58			Urticaria		Antihistamine	Urticaria		Cortisone and Antihistamine				Comirnaty	Comirnaty	
30	F	68	Yes (drugs)					Erythema						Janssen	Comirnaty	
31	F	25		PEG		Itching	Antihistamine							Comirnaty	Comirnaty	
32	F	39		PEG					Urticaria	Antihistamine				Comirnaty	Comirnaty	Comirnaty
33	F	71	Yes (contrast medium)			Urticaria								Comirnaty	Comirnaty	
34	F	56				Urticaria								Comirnaty		
35	F	58	Yes (drugs)			Urticaria	Cortisone							Comirnaty		
36	F	22				Asthma								Comirnaty		
37	F	32			Urticaria									Comirnaty		
38	F	66				Asthma								Comirnaty	Comirnaty	
39	M	53				Erythema								Spikevax	Spikevax	
40	M	52				Erythema	Antihistamine							Spikevax	Spikevax	
41	F	49	Yes (drugs, food, contrast medium)		Angioedema			Angioedema						Comirnaty	Comirnaty	
42	F	78	Yes (drugs)						Itching	Antihistamine				Comirnaty	Comirnaty	
43	M	61				Urticaria								Comirnaty	Comirnaty	
44	F	59			Urticaria			Urticaria						Comirnaty	Comirnaty	Comirnaty
45	F	38							Erythema					Comirnaty	Comirnaty	Comirnaty
46	F	69				Urticaria	Cortisone		Urticaria	Cortisone				Comirnaty	Comirnaty	Comirnaty
47	F	27							Erythema	Antihistamine				Comirnaty	Comirnaty	
48	F	73			Erythema		Antihistamine							Vaxzevria	Vaxzevria	
49	F	55				Angioedema	Cortisone		Angioedema	Cortisone				Comirnaty	Comirnaty	
50	F	60				Urticaria	Antihistamine		Urticaria, Asthma	Cortisone and Antihistamine				Spikevax	Spikevax	
51	M	58							Urticaria	Antihistamine				Comirnaty	Comirnaty	
52	F	60			Urticaria									Spikevax		
53	F	54		PEG and PS80		Erythema								Spikevax		
54	F	22						Urticaria		Antihistamine				Comirnaty	Comirnaty	
55	F	37				Asthma	Cortisone							Comirnaty		
56	F	51	Yes (drugs)						Asthma	Cortisone				Comirnaty	Comirnaty	Comirnaty
57	F	42		PEG	Urticaria		Cortisone and antihistamine		Oculorinitis					Spikevax	Comirnaty	
58	F	56		PEG	Erythema		Cortisone							Comirnaty	Novavax	
59	F	64		PEG	Angioedema									Janssen	Comirnaty	
60	F	60	Yes (drugs)	PEG					Asthma					Comirnaty	Comirnaty	
61	M	28	Yes (drugs)	PEG		Urticaria								Comirnaty		
62	F	57				Urticaria								Comirnaty	Comirnaty	
63	F	49				Urticaria			Urticaria					Comirnaty	Comirnaty	

Reactions only to dose I (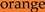): N = 29. Reactions to doses I and II (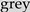): N = 17. Reactions only to dose II (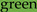): N = 11. Reactions to doses II and III (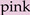): N = 2. Reactions only to dose III (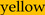): N = 3. Reactions to doses I and III (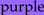): N = 1.

**Table 4:** COVID-19 vaccines administered to the general population of ASUGI (Trieste–Gorizia catchment area) and adverse reactions reported by 31 December 2021. Number (N) and percentage of adverse reactions out of total vaccine doses.

	I Dose (N)	II Dose (N)	III Dose (N)	Total Doses (N)	Total Adverse Reactions N (%)	Allergic Reactions * N (%)
**Vaxzevria** (AstraZeneca)	45,873	42,735	-	88,608	370 (0.41)	64 (0.07)
**Comirnaty** (Pfizer BioNTech)	215,426	201,266	98,510	515,202	1571 (0.30)	223 (0.043)
**Spikevax** (Moderna)	42,670	33,216	26,013	101,899	183 (0.18)	44 (0.043)
**Janssen** (Johnson & Johnson)	6936				15 (0.22)	2 (0.029)
**Unreported**	593	255	16	864	-	
**TOTAL**	**311,498**	**277,472**	**124,539**	**706,573**	**2139 (0.30)**	**333 (0.047)**

* urticaria, angioedema, itching, asthma and rhinitis. Thirty-three cases were classified as “severe reactions” requiring treatment and medical support. No anaphylaxis was recorded.

## Data Availability

The datasets generated and analyzed during the current study are not publicly available, since they were purposively collected by the authors for the present study, but they may be available from the corresponding author on reasonable request.

## Supplementary Materials

The following supporting information can be downloaded at: https://www.mdpi.com/article/10.3390/vaccines10101616/s1, Supplementary Table S1: Definition of the main immunological reactions, IgE mediated and not.

## Abbreviations

PEG = polyethylene glycol; PS80 = polysorbate 800; ATF = allergy task force; VAERS = Vaccine Adverse Event Reporting System; CARPA = complement activation-related pseudoallergy; HCWs = health care workers.
