# Supplementary material for: Allergic Reactions to COVID-19 Vaccination in High-Risk Allergic Patients: The Experience of Trieste University Hospital (North-Eastern Italy)
Source: Vaccines (Basel). 2022 Sep 27;10(10):1616. doi: 10.3390/vaccines10101616 (PMC9607499; doi:10.3390/vaccines10101616)
Supplement: Supplementary file 1 [file vaccines-10-01616-s001.zip › vaccines-1896522-supplementary.pdf]

Supplementary Table S1. Definition of the main immunological reactions, IgE mediated and not.

REACTION	DEFINITION
**Allergy**	Hypersensitivity to a substance, causing the body to react to any contact with that substance. Individuals are considered to have clinically significant allergy or allergic disease when they have both allergen-specific IgE and develop symptoms following exposure to the allergen [13]
**Atopy**	Genetic predisposition to exaggerated immune response to allergens, leading to CD4+ Th2 differentiation and overproduction of IgE [13]
**Sensitization**	Production of allergen-specific IgE, not necessarily resulting in allergic reaction since individuals may produce IgE to allergens in a given substance, without developing symptoms upon exposure to that substance. The reason why some individuals are only sensitized whereas others develop active allergic disease is still unclear [13]
**Hypersensitivity**	Unintended, exaggerated or inappropriate response of the immune or inflammatory cells against harmless antigens (also by a medication of vaccine), potentially causing consequent tissue damage [14]
**Asthma**	Chronic inflammatory disease of the airways, associated with hypersensitivity and airway hyper-responsiveness to allergens or physical exercise, leading to recurrent symptoms due to airflow obstruction as wheezing, dyspnea, cough, chest tightness [15 ,16]
**Allergic rhinitis**	Common long-standing disorder (often undetected) strongly associate with asthma and conjunctivitis. Classic symptoms include nasal congestion, nasal itch, rhinorrhea and sneezing [15,16]
**Anaphylaxis**	A severe allergic reaction. Symptoms start very quickly (usually within minutes) and almost always within 4 h of vaccination and typically include hives; swelling of mouth, lips, tongue, or throat, shortness of breath, wheezing, or chest tightness; or low blood pressure or loss of consciousness [15,17]
**Anaphylactoid reaction**	Severe systemic reactions clinically identical to anaphylaxis, not mediated by over-production of IgE, but through direct activation of complement or non-immune-mediated release of mediators from mast cells and/or basophils [17]
**Angioedema**	Acute temporary swelling beneath skin or mucosae (lip, tongue, throat, other) [15]
**Atopic dermatitis**	Chronic, highly pruritic inflammatory skin disease, resulting from interplay between defects in skin barrier function, environmental and infectious agents, and immune abnormalities [15]
**Contact dermatitis**	Erythemato-papulous persistent skin rash 2–3 days since contact or exposure to an antigen [16]
**Urticaria**	Transient weal and flare reaction of several skin sites, typically accompanied with itching and usually resolving within 24 hours. Acute urticaria refers to lesions occurring < 6 weeks, whereas chronic urticaria to lesions occurring > 6 weeks. Urticarial lesions usually are present most days of the week [15,16]
